# Avelumab first-line maintenance plus best supportive care (BSC) vs BSC alone for advanced urothelial carcinoma: JAVELIN Bladder 100 Japanese subgroup analysis

**DOI:** 10.1007/s10147-021-02067-8

**Published:** 2022-01-01

**Authors:** Yoshihiko Tomita, Yoshiaki Yamamoto, Norihiko Tsuchiya, Hiroomi Kanayama, Masatoshi Eto, Hideaki Miyake, Thomas Powles, Mizuki Yoshida, Yuichiro Koide, Yoshiko Umeyama, Alessandra di Pietro, Hirotsugu Uemura

**Affiliations:** 1 Department of Urology and Department of Molecular Oncology, Niigata University Graduate School of Medicine, 1-757 Asahimachi, Chuou-ku, Niigata, 951-8510 Japan; 2 Department of Urology, Graduate School of Medicine, Yamaguchi University, 1-1-1 Minami-Kogushi, Ube, Yamaguchi 755-8505 Japan; 3 Department of Urology, Yamagata University Faculty of Medicine, 2-2-2 Iida-Nishi, Yamagata, 990-9585 Japan; 4 Department of Urology, Tokushima University Graduate School of Biomedical Sciences, 3-18-15, Kuramoto-cho, Tokushima, 770-8503 Japan; 5 Department of Urology, Kyushu University Graduate School of Medical Sciences, 3-1-1 Maidashi, Higashi-ku, Fukuoka, 812-8582 Japan; 6 Department of Urology, Hamamatsu University School of Medicine, 1-20-1 Handayama, Higashi-ku, Hamamatsu, Shizuoka 431-3192 Japan; 7 Experimental Cancer Medicine Centre, Barts Cancer Institute, Queen Mary University of London, St Bartholomew’s Hospital, West Smithfield, London, EC1A 7BE UK; 8 Pfizer R&D Japan, 3-22-7 Yoyogi, Shibuya-ku, Tokyo, 151-8589 Japan; 9 Pfizer srl, Via Anna Maria Mozzoni, 12, 20152 Milan, Italy; 10 Department of Urology, Faculty of Medicine, Kindai University, 377-2 Ohno-Higashi, Osakasayama, Osaka 589-8511 Japan

**Keywords:** Avelumab, Immunotherapy, Maintenance, Japan, Urinary bladder neoplasms

## Abstract

**Background:**

The phase 3 JAVELIN Bladder 100 trial showed significantly prolonged overall survival (OS) with avelumab as first-line (1L) maintenance therapy + best supportive care (BSC) vs BSC alone in patients with advanced urothelial carcinoma (UC) that had not progressed with 1L platinum-containing chemotherapy. Efficacy and safety were assessed in patients enrolled in Japan.

**Methods:**

Patients with locally advanced or metastatic UC that had not progressed with 4–6 cycles of 1L platinum-containing chemotherapy were randomized to avelumab (10 mg/kg intravenously every 2 weeks) + BSC or BSC alone. The primary endpoint was OS, and secondary endpoints included progression-free survival (PFS) and safety.

**Results:**

In Japanese patients (*n* = 73) randomized to avelumab + BSC (*n* = 36) or BSC alone (*n* = 37), median OS was 24.7 months (95% CI, 18.2-not estimable) vs 18.7 months (95% CI, 12.8–33.0), respectively (HR, 0.81 [95% CI, 0.41–1.58]), and median PFS was 5.6 months (95% CI, 1.9–9.4) vs 1.9 months (95% CI, 1.9–3.8), respectively (HR, 0.63 [95% CI, 0.36–1.11]). In the avelumab + BSC and BSC-alone arms, grade ≥ 3 treatment-emergent adverse events (AEs) occurred in 50.0% vs 8.1%, including grade ≥ 3 treatment-related AEs in 13.9% vs 0%, respectively. Efficacy and safety results in Japanese patients were generally consistent with findings in the overall trial population.

**Conclusion:**

Avelumab 1L maintenance treatment showed a favorable benefit-risk balance in Japanese patients, supporting avelumab 1L maintenance as a new standard of care in Japanese patients with advanced UC that has not progressed with 1L platinum-containing chemotherapy.

**Trial registration:**

Clinicaltrials.gov NCT02603432.

**Supplementary Information:**

The online version contains supplementary material available at 10.1007/s10147-021-02067-8.

## Introduction

Urothelial carcinoma (UC), which originates in the cells lining the bladder or other parts of the urothelial tract, is one of the most common cancers; bladder cancer itself is the 11th most common cancer globally and the 13th most common cancer in Japan [1, 2]. Platinum-based chemotherapy is a standard first-line (1L) treatment for eligible patients with advanced UC [3, 4]. Although most patients (≈ 75%) will respond or have disease control initially, long-term benefits are limited and overall survival (OS) is generally short (median, ≈ 9–14 months) [5–7].

Immune checkpoint inhibitors that target programmed death 1 (PD-1) or programmed death ligand 1 (PD-L1) have shown clinical activity in patients with advanced UC [3]. Consequently, several immune checkpoint inhibitors have been approved in various countries for the treatment of patients with advanced UC that has progressed during or following platinum-containing chemotherapy and in some countries for 1L treatment of cisplatin-ineligible patients with PD-L1 + tumors [3, 8]. Avelumab is an anti–PD-L1 antibody [9–11]. In Japan, avelumab has been approved as monotherapy for patients with Merkel cell carcinoma. It has also been shown that the combination of avelumab plus axitinib is efficacious and tolerable in Japanese patients with treatment-naive advanced renal cell carcinoma [12], resulting in approval in this indication in Japan.

The phase 3 JAVELIN Bladder 100 trial investigated avelumab as 1L maintenance therapy in patients with locally advanced or metastatic UC that had not progressed with 1L platinum-containing chemotherapy [13]. The trial met its primary objective, demonstrating significantly prolonged OS with avelumab + best supportive care (BSC) vs BSC alone in all randomized patients (hazard ratio [HR], 0.69 [95% CI, 0.56–0.86]; *P* = 0.001) and patients with PD-L1 + tumors (HR, 0.56 [95% CI, 0.40–0.79]; *P* < 0.001). Results from the trial supported the approval of avelumab 1L maintenance in several countries, including in Japan, as well as its inclusion in international treatment guidelines as a new standard of care for advanced UC in cisplatin-eligible and cisplatin-ineligible patients [3, 14, 15], including guidelines developed by the Japanese Urological Association [16]. Here, we report a post hoc subgroup analysis of the JAVELIN Bladder 100 trial in patients enrolled in Japan.

## Materials and methods

### Study design and patients

JAVELIN Bladder 100 (NCT02603432) was an international, open-label, phase 3 trial. The study design has been reported in detail previously [13]. Briefly, the study enrolled patients who had histologically confirmed, unresectable locally advanced or metastatic UC; had no disease progression after receiving 4–6 cycles of 1L chemotherapy with cisplatin + gemcitabine or carboplatin + gemcitabine; had a treatment-free interval of 4–10 weeks since the last dose of chemotherapy; were aged ≥ 18 years (≥ 20 years in Japan); and had an ECOG performance status (ECOG PS) of 0 or 1.

Patients were randomly assigned (1:1) to receive either maintenance therapy with avelumab plus BSC (avelumab arm) or BSC alone (control arm). Allocation was stratified by best response to 1L chemotherapy (complete response [CR] or partial response [PR] vs stable disease) and by metastatic site when 1L chemotherapy was initiated (visceral vs non-visceral). The non-visceral stratum included both patients with unresectable locally advanced disease or only non-visceral disease, including bone metastasis.

The study was conducted in accordance with the ethics principles of the Declaration of Helsinki and Good Clinical Practice guidelines, defined by the International Council for Harmonisation. The protocol, amendments, and informed consent forms were approved by the institutional review board or independent ethics committee at each trial site, and all patients provided written consent.

### Outcomes

The primary endpoint was OS, assessed both in the overall population (all randomized patients) and in patients with PD-L1 + tumors (PD-L1 + population). Secondary endpoints included: progression-free survival (PFS; time from randomization to the date of the first documentation of progressive disease [PD] or death due to any cause, whichever occurs first); objective response (confirmed CR or PR); time to response (time from randomization to the first documentation of objective response) and duration of response (time from the first documentation of objective response to the first documentation of PD or death due to any cause, whichever occurs first) in patients with the objective response; disease control (CR, PR, non-CR/non-PD, or stable disease for ≥ 6 weeks); and safety. All endpoints were measured after randomization (after chemotherapy), and all tumor assessments were performed according to RECIST version 1.1 with a blinded independent central review (BICR).

### Treatment and assessments

Patients in the avelumab arm were treated with avelumab 10 mg/kg as a 1-h intravenous infusion every 2 weeks, and all patients also received BSC; patients in the control arm received BSC alone. BSC (e.g., antibiotics, nutritional support, hydration, or pain management) was administered per local practice based on patient needs and clinical judgment; other systemic antitumor therapy was not permitted, but palliative local radiotherapy for isolated lesions was acceptable.

Tumor assessments were performed according to RECIST version 1.1 every 8 weeks for 12 months, then every 12 weeks thereafter, until disease progression was confirmed by BICR. Adverse events (AEs) were graded according to the National Cancer Institute Common Terminology Criteria for AEs, version 4.03. PD-L1 expression was assessed in tumor samples using the Ventana PD-L1 immunohistochemistry assay (SP263; Ventana Medical Systems). PD-L1 + status was defined as ≥ 1 of the following: ≥ 25% of tumor cells stained for PD-L1, ≥ 25% of immune cells stained for PD-L1 if > 1% of the tumor area contained immune cells, or 100% of immune cells stained for PD-L1 if ≤ 1% of the tumor area contained immune cells.

### Statistical analysis

The Japanese subgroup comprised all randomized patients enrolled at sites in Japan. Efficacy endpoints were assessed for the Japanese subgroup according to the intention-to-treat principle. OS and PFS were estimated using the Kaplan–Meier method, and HRs and associated 95% CIs for OS and PFS were calculated using an unstratified Cox proportional hazards model. Objective response rate and disease control rate were calculated by treatment group, and exact 2-sided 95% CIs were calculated using the Clopper–Pearson method. Safety was assessed in all patients who received ≥ 1 dose of avelumab in the avelumab arm and in all the patients who completed the cycle 1 day 1 visit in the control arm. AEs were summarized using Medical Dictionary for Regulatory Activities (version 22.1) preferred terms. Statistical analyses were conducted using SAS, version 9.4. Data from a preplanned interim analysis are reported; however, because the primary endpoints were met, this represents the final analysis. The data cutoff date for all analyses was October 21, 2019.

## Results

### Patient characteristics and disposition

Details of all patients enrolled in the JAVELIN Bladder 100 trial have been reported previously [13]. Overall, 700 patients were randomized to receive avelumab maintenance therapy + BSC (avelumab arm; *n* = 350) or BSC alone (control arm; *n* = 350). In total, 73 patients were enrolled in Japan; 36 and 37 were randomized to the avelumab and control arms, respectively. In the overall population, 51.1% of patients had a PD-L1 + tumor, including 57.5% of patients in the Japanese subgroup.

Demographics and baseline characteristics were generally balanced between the treatment arms (Table 1). Differences noted between the Japanese subgroup and the overall population included (avelumab/control arm): lower median weight (62.9/61.9 kg vs 72.4/73.0 kg), lower proportion with an ECOG PS of ≥ 1 (16.7%/10.8% vs 39.1%/39.7%), higher proportion with an upper tract primary tumor (58.3%/56.8% vs 30.3%/23.1%), and slightly higher proportion with baseline creatinine clearance < 60 mL/min (58.3%/51.4% vs 48.0%/42.3%). Furthermore, in Japanese patients with PD-L1 + tumors, differences in demographics and baseline characteristics between the avelumab and control arms included: higher proportion with an ECOG PS of ≥ 1 (21.1% vs 4.3%; Table 1), lower proportion with visceral metastases (26.3% vs 47.8%; Table 1), higher proportion with baseline creatinine clearance < 60 mL/min (63.2% vs 39.1%; Table 1), and lower proportion who had an objective response to 1L chemotherapy (52.6% vs 65.2%; Table 2).

**Table 1:** Demographics and baseline characteristics of the Japanese subgroup and the overall population. Source: Data for the overall population have been published previously in Ref. [13]. Copyright © 2020 Massachusetts Medical Society. Reprinted with permission

	Japanese subgroup				Overall population [13]			
	Randomised patients (*n* = 73)		PD-L1 + population (*n* = 42)		Randomised patients (*N* = 700)		PD-L1 + population (*N* = 358)	
	Avelumab + BSC (*n* = 36)	BSC (*n* = 37)	Avelumab + BSC (*n* = 19)	BSC (*n* = 23)	Avelumab + BSC (*n* = 350)	BSC (*n* = 350)	Avelumab + BSC (*n* = 189)	BSC (*n* = 169)
Age, median (range), years	70.5 (46–84)	69 (43–83)	71 (55–84)	69 (49–83)	68 (37–90)	69 (32–89)	70 (37–90)	70 (32–84)
Weight, median (range), kg	62.9 (41.9–100.6)	61.9 (37.2–86.8)	62.8 (44.8–100.6)	62.8 (37.2–86.8)	72.4 (40.0–136.0)	73.0 (37.2–135.3)	73.5 (40.0–136.0)	73.5 (37.2–131.8)
Sex, *n* (%)								
Male	25 (69.4)	26 (70.3)	12 (63.2)	16 (69.6)	266 (76.0)	275 (78.6)	145 (76.7)	129 (76.3)
Female	11 (30.6)	11 (29.7)	7 (36.8)	7 (30.4)	84 (24.0)	75 (21.4)	44 (23.3)	40 (23.7)
Race, *n* (%)								
White	0	0	0	0	232 (66.3)	238 (68.0)	121 (64.0)	119 (70.4)
Asian	36 (100)	37 (100)	19 (100)	23 (100)	75 (21.4)	81 (23.1)	42 (22.2)	33 (19.5)
Black/African American	0	0	0	0	2 (0.6)	0	1 (0.5)	0
Other	0	0	0	0	21 (6.0)	15 (4.3)	12 (6.3)	7 (4.1)
Unknown	0	0	0	0	20 (5.7)	16 (4.6)	13 (6.9)	10 (5.9)
Pooled geographic region, *n* (%)								
North America	0	0	0	0	12 (3.4)	22 (6.3)	8 (4.2)	8 (4.7)
Europe	0	0	0	0	214 (61.1)	203 (58.0)	110 (58.2)	102 (60.4)
Asia	36 (100)	37 (100)	19 (100)	23 (100)	73 (20.9)	74 (21.1)	40 (21.2)	31 (18.3)
Australasia	0	0	0	0	34 (9.7)	37 (10.6)	20 (10.6)	24 (14.2)
Rest of the world	0	0	0	0	17 (4.9)	14 (4.0)	11 (5.8)	4 (2.4)
ECOG performance status, *n* (%)								
0	30 (83.3)	33 (89.2)	15 (78.9)	22 (95.7)	213 (60.9)	211 (60.3)	114 (60.3)	107 (63.3)
1	5 (13.9)	4 (10.8)	3 (15.8)	1 (4.3)	136 (38.9)	136 (38.9)	74 (39.2)	61 (36.1)
2	1 (2.8)	0	1 (5.3)	0	1 (0.3)	0	1 (0.5)	0
3	0	0	0	0	0	3 (0.9)	0	1 (0.6)
Site of primary tumor, *n* (%)^a^								
Upper tract	21 (58.3)	21 (56.8)	10 (52.6)	13 (56.5)	106 (30.3)	81 (23.1)	44 (23.3)	35 (20.7)
Lower tract	15 (41.7)	16 (43.2)	9 (47.4)	10 (43.5)	244 (69.7)	269 (76.9)	145 (76.7)	134 (79.3)
Site of baseline metastasis before chemotherapy, *n* (%)								
Visceral	17 (47.2)	19 (51.4)	5 (26.3)	11 (47.8)	191 (54.6)	191 (54.6)	88 (46.6)	79 (46.7)
Non-visceral^b^	19 (52.8)	18 (48.6)	14 (73.7)	12 (52.2)	159 (45.4)	159 (45.4)	101 (53.4)	90 (53.3)
Creatinine clearance, *n* (%)								
≥ 60 mL/min	15 (41.7)	16 (43.2)	7 (36.8)	12 (52.2)	181 (51.7)	196 (56.0)	104 (55.0)	97 (57.4)
< 60 mL/min	21 (58.3)	19 (51.4)	12 (63.2)	9 (39.1)	168 (48.0)	148 (42.3)	84 (44.4)	70 (41.4)
Unknown	0	2 (5.4)	0	2 (8.7)	1 (0.3)	6 (1.7)	1 (0.5)	2 (1.2)
PD-L1 status, *n* (%)								
Positive	19 (52.8)	23 (62.2)	19 (100)	23 (100)	189 (54.0)	169 (48.3)	189 (100)	169 (100)
Negative	15 (41.7)	9 (24.3)	0	0	139 (39.7)	131 (37.4)	0	0
Unknown	2 (5.6)	5 (13.5)	0	0	22 (6.3)	50 (14.3)	0	0

*BSC* best supportive care, *PD-L1* programmed death ligand 1

^a^The upper tract was defined as the renal pelvis or ureter; the lower tract was defined as the bladder, urethra, or prostate gland

^b^Non-visceral includes patients with locally advanced disease or only non-visceral disease, including bone metastasis

**Table 2:** Summar﻿y of first-line chemotherapy received by patients in the Japanese subgroup and the overall population. Source: Data for the overall population have been published previously in Ref. [13]. Copyright © 2020 Massachusetts Medical Society. Reprinted with permission

	Japanese subgroup				Overall population [13]			
	Randomized patients (*n* = 73)		PD-L1 + population (*n* = 42)		Randomized patients (*N* = 700)		PD-L1 + population (*N* = 358)	
	Avelumab + BSC (*n* = 36)	BSC (*n* = 37)	Avelumab + BSC (*n* = 19)	BSC (*n* = 23)	Avelumab + BSC (*n* = 350)	BSC (*n* = 350)	Avelumab + BSC (*n* = 189)	BSC (*n* = 169)
1L chemotherapy regimen, *n* (%)								
Gemcitabine + cisplatin	25 (69.4)	29 (78.4)	14 (73.7)	18 (78.3)	183 (52.3)	206 (58.9)	101 (53.4)	98 (58.0)
Gemcitabine + carboplatin	9 (25.0)	8 (21.6)	4 (21.1)	5 (21.7)	147 (42.0)	122 (34.9)	74 (39.2)	54 (32.0)
Gemcitabine + cisplatin or carboplatin^a^	2 (5.6)	0	1 (5.3)	0	20 (5.7)	20 (5.7)	14 (7.4)	15 (8.9)
Not reported	0	0	0	0	0	2 (0.6)	0	2 (1.2)
Best response to 1L chemotherapy, *n* (%)								
Complete or partial response	22 (61.1)	22 (59.5)	10 (52.6)	15 (65.2)	253 (72.3)	252 (72.0)	139 (73.5)	128 (75.7)
Stable disease	14 (38.9)	15 (40.5)	9 (47.4)	8 (34.8)	97 (27.7)	98 (28.0)	50 (26.5)	41 (24.3)
Cisplatin, duration of treatment								
*n*	27	29	15	18	203	226	115	113
Median (range), weeks	16.7 (3.0–37.6)	18.0 (12.0–54.1)	15.9 (7.0–37.6)	18.9 (12.0–54.1)	18.0 (3.0–37.6)	18.0 (3.0–82.9)	18.0 (3.0–37.6)	18.0 (3.0–54.1)
Carboplatin, duration of treatment								
* n*	11	8	5	5	167	142	88	69
Median (range), weeks	15.6 (8.9–23.1)	15.6 (13.3–18.7)	13.9 (12.4–23.1)	14.1 (13.3–18.7)	17.0 (3.0–39.9)	16.1 (3.0–36.1)	17.3 (3.0–24.7)	14.0 (3.0–22.1)
Gemcitabine, duration of treatment								
* n*	36	37	19	23	350	348	189	167
Median (range), weeks	17.3 (10.9–38.4)	18.9 (12.6–55.3)	17.1 (13.0–38.4)	18.9 (12.6–55.3)	19.0 (9.0–39.9)	19.0 (9.9–82.9)	19.0 (9.0–38.4)	18.6 (9.9–55.3)

*1L* first-line, *BSC* best supportive care, *PD-L1* programmed death ligand 1

^a^Includes patients who switched platinum-based regimens while receiving 1L chemotherapy

Differences in prior 1L chemotherapy were observed between the Japanese subgroup and the overall population (Table 2), including (avelumab/control arm): higher proportion who had received 1L gemcitabine + cisplatin (69.4%/78.4% vs 52.3%/58.9%) and lower proportion who had an objective response to 1L chemotherapy (61.1%/59.5% vs 72.3%/72.0%). Median durations of 1L chemotherapy in the Japanese subgroup and overall population in the avelumab/control arm were 16.7/18.0 vs 18.0/18.0 weeks for cisplatin, 15.6/15.6 vs 17.0/16.1 weeks for carboplatin, and 17.3/18.9 vs 19.0/19.0 weeks for gemcitabine.

At the data cutoff date, 7 of 36 (19.4%) Japanese patients in the avelumab arm and 2 of 37 (5.4%) in the control arm remained on study treatment (Online Resource 1). Of patients who had discontinued from the avelumab arm (29 [80.6%]) or control arm (35 [94.6%]), reasons for treatment discontinuation were: disease progression (21 [58.3%] vs 28 [75.7%]), AE (4 [11.1%] vs 0), death (2 [5.6%] vs 0), withdrawal by patient (1 [2.8%] vs 6 [16.2%]), physician’s decision (1 [2.8%] vs 0), and overall health deterioration (0 vs 1 [2.7%]).

### Efficacy

**Fig. 1:**
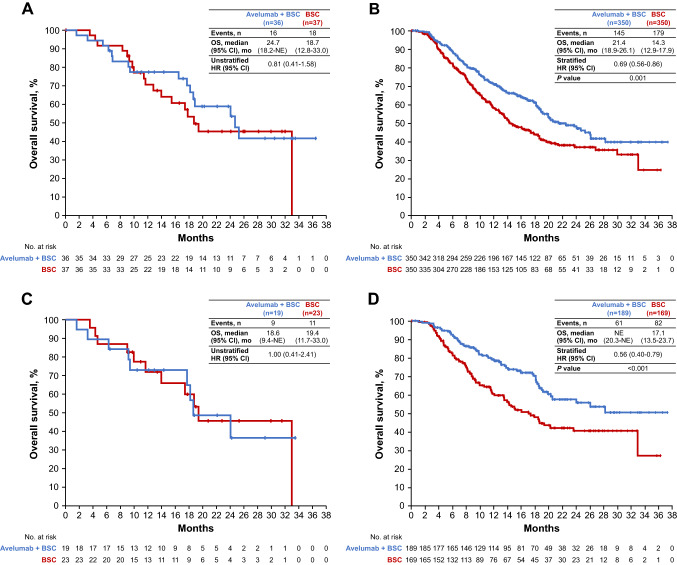
OS in **A** the Japanese subgroup, **B** the overall population [13], **C** Japanese patients with PD-L1 + tumors, and **D** all patients with PD-L1 + tumors [13]. *BSC* best supportive care, *HR* hazard ratio, *NE* not estimable, *OS* overall survival, *PD-L1* programmed death ligand 1. At data cutoff (October 21, 2019), the median follow-up for OS in all Japanese patients was ≥ 24 months in both arms. **B** and **D** From Powles et al. [13]. Copyright © 2020 Massachusetts Medical Society. Reprinted with permission

Efficacy results in the Japanese subgroup were generally consistent with those in the overall population. Median follow-up for OS in the Japanese subgroup was 24.2 months (95% CI, 18.8–31.8) in the avelumab arm vs 24.1 months (95% CI, 17.8–28.1) in the control arm. OS in both the Japanese subgroup and overall population are shown in Fig. 1. In the Japanese subgroup, median OS was 24.7 months (95% CI, 18.2-not estimable) in the avelumab arm vs 18.7 months (95% CI, 12.8–33.0) in the control arm (HR, 0.81 [95% CI, 0.41–1.58]) (Fig. 1A). In Japanese patients with PD-L1 + tumors, median OS was 18.6 months (95% CI, 9.4-not estimable) in the avelumab arm vs 19.4 months (95% CI, 11.7–33.0) in the control arm (HR, 1.00 [95% CI, 0.41–2.41]) (Fig. 1C).

**Fig. 2:**
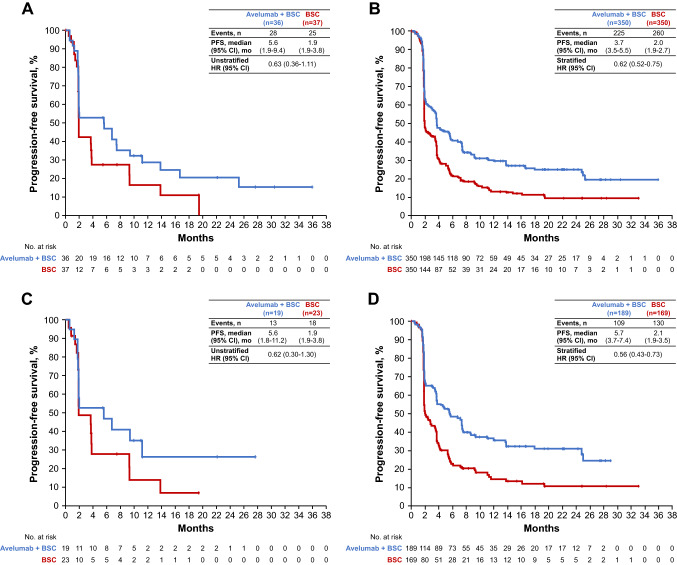
PFS by BICR in **A** the Japanese subgroup, **B** the overall population [13], **C** Japanese patients with PD-L1 + tumors, and **D** all patients with PD-L1 + tumors [13]. *BICR* blinded independent central review, *BSC* best supportive care, *HR* hazard ratio, *PD-L1* programmed death ligand 1, *PFS* progression-free survival. **B** and **D** From Powles et al. [13]. Copyright © 2020 Massachusetts Medical Society. Reprinted with permission

PFS in both the Japanese subgroup and overall population are shown in Fig. 2. Median PFS (by BICR) in Japanese patients was 5.6 months (95% CI, 1.9–9.4) in the avelumab arm vs 1.9 months (95% CI, 1.9–3.8) in the control arm (HR, 0.63 [95% CI, 0.36–1.11]) (Fig. 2A). In Japanese patients with PD-L1 + tumors, median PFS was 5.6 months (95% CI, 1.8–11.2) in the avelumab arm vs 1.9 months (95% CI, 1.9–3.8) in the control arm (HR, 0.62 [95% CI, 0.30–1.30]) (Fig. 2C).

The objective response rates (by BICR) in the Japanese subgroup in the avelumab and control arms were 5.6% (95% CI, 0.7–18.7) vs 0% (95% CI, 0–9.5), respectively (Table 3), including 1 CR and 1 PR in the avelumab arm. In the 2 responding patients, time to response was 2.0 and 5.6 months, and duration of response was 34.0 months (ongoing at data cutoff) and 8.3 months, respectively.

**Table 3:** Confirmed objective response per BICR in the Japanese subgroup and the overall population. Source: Data for the overall population have been published previously in Ref. [13]. Copyright © 2020 Massachusetts Medical Society. Reprinted with permission

	Japanese subgroup (*n* = 73)		Overall population (*N* = 700) [13]	
	Avelumab + BSC (*n* = 36)	BSC (*n* = 37)	Avelumab + BSC (*n* = 350)	BSC (*n* = 350)
Confirmed best overall response, *n* (%)				
Complete response	1 (2.8)	0	21 (6.0)	3 (0.9)
Partial response	1 (2.8)	0	13 (3.7)	2 (0.6)
Stable disease	3 (8.3)	6 (16.2)	44 (12.6)	46 (13.1)
Noncomplete response/nonprogressive disease	7 (19.4)	4 (10.8)	66 (18.9)	45 (12.9)
Progressive disease	17 (47.2)	17 (45.9)	130 (37.1)	169 (48.3)
Not evaluable	7 (19.4)	10 (27.0)	76 (21.7)	85 (24.3)
Objective response, *n* (%)	2 (5.6)	0	34 (9.7)	5 (1.4)
(95% CI)	(0.7–18.7)	(0–9.5)	(6.8–13.3)	(0.5–3.3)
Disease control, *n* (%)	12 (33.3)	10 (27.0)	144 (41.1)	96 (27.4)
(95% CI)	(18.6–51.0)	(13.8–44.1)	(35.9–46.5)	(22.8–32.4)

*BICR* blinded independent central review, *BSC* best supportive care

### Subsequent therapy

Compared with the overall population, a higher proportion of Japanese patients received a subsequent anticancer drug therapy after discontinuing study therapy (Table 4). In Japanese patients in the avelumab vs control arms, a subsequent anticancer drug therapy was received by 61.1% vs 81.1%, respectively (compared with 42.3% vs 61.7%, respectively, in the overall population). This included a PD-1 or PD-L1 inhibitor in 38.9% vs 67.6% of the avelumab vs control arms, respectively (compared with 6.3% vs 43.7%, respectively, in the overall population). In the Japanese subgroup, the most commonly received subsequent anticancer drug therapies (avelumab vs control arm, respectively) were pembrolizumab (38.9% vs 64.9%), gemcitabine (36.1% vs 43.2%), carboplatin (30.6% vs 21.6%), cisplatin (25.0% vs 29.7%), and paclitaxel (13.9% vs 16.2%) (Online Resource 2).

**Table 4:** Subsequent anticancer drug therapy in the Japanese subgroup and the overall population. Source: Data for the overall population have been published previously in Ref. [13]. Copyright © 2020 Massachusetts Medical Society. Reprinted with permission

	Japanese subgroup				Overall population [13]			
	Randomised patients (*n* = 73)		PD-L1 + population (*n* = 42)		Randomised patients (*N* = 700)		PD-L1 + population (*N* = 358)	
	Avelumab + BSC (*n* = 36)	BSC (*n* = 37)	Avelumab + BSC (*n* = 19)	BSC (*n* = 23)	Avelumab + BSC (*n* = 350)	BSC (*n* = 350)	Avelumab + BSC (*n* = 189)	BSC (*n* = 169)
Discontinued and received subsequent drug therapy, *n* (%)^a^	22 (61.1)	30 (81.1)	9 (47.4)	19 (82.6)	148 (42.3)	216 (61.7)	68 (36.0)	109 (64.5)
PD-L1/PD-1 inhibitor	14 (38.9)	25 (67.6)	6 (31.6)	16 (69.6)	22 (6.3)	153 (43.7)	10 (5.3)	81 (47.9)
Fibroblast growth factor receptor inhibitor	1 (2.8)	0	1 (5.3)	0	9 (2.6)	8 (2.3)	3 (1.6)	4 (2.4)
Any other drug	19 (52.8)	20 (54.1)	8 (42.1)	10 (43.5)	140 (40.0)	119 (34.0)	67 (35.4)	57 (33.7)
Discontinued with no subsequent drug therapy, *n* (%)	7 (19.4)	5 (13.5)	5 (26.3)	2 (8.7)	117 (33.4)	108 (30.9)	63 (33.3)	47 (27.8)
Study treatment ongoing, *n* (%)	7 (19.4)	2 (5.4)	5 (26.3)	2 (8.7)	85 (24.3)	26 (7.4)	58 (30.7)	13 (7.7)

*BSC* best supportive care, *PD-1* programmed death 1, *PD-L1* programmed death ligand 1

^a^Some patients received > 1 category of subsequent therapy

### Safety

At data cutoff, the median duration of treatment in Japanese patients was 32.0 weeks (range, 2.0–159.9) in the avelumab arm and 9.1 weeks (range, 0.1–90.0) in the control arm (Online Resource 3). The median avelumab dose intensity was 17.6 mg/kg/4-week cycle (range, 10.0–19.8), with a median relative dose intensity of 87.8% (range, 50.0–99.1), similar to that in the overall population.

The safety profile of avelumab in Japanese patients was generally consistent with that in the overall population (Table 5), with slight differences in rates of some treatment-emergent AEs (TEAEs; related or unrelated to treatment) (Table 6). TEAEs of any grade occurred in all Japanese patients in the avelumab arm and in 56.8% of patients in the control arm, including grade ≥ 3 TEAEs in 50.0% and 8.1% of patients, respectively. In the avelumab arm, the most common TEAEs of any grade were pyrexia (27.8%), anemia (19.4%), and nasopharyngitis (19.4%); the most common grade ≥ 3 TEAEs were anemia (11.1%), pyelonephritis (5.6%), increased amylase (5.6%), and increased blood triglycerides (5.6%). TEAEs led to interruption of avelumab in 16 patients (44.4%; most commonly due to pyelonephritis in 3 patients [8.3%], hyperthyroidism in 2 patients [5.6%]) and discontinuation of avelumab in 4 patients (11.1%; 1 case each due to anemia, myocardial infarction, gastric ulcer, sepsis, platelet count decreased, and interstitial lung disease).

**Table 5:** Safety summary in the Japanese subgroup and the overall population. Source: Data for the overall population have been published previously in Ref. [13]. Copyright © 2020 Massachusetts Medical Society. Reprinted with permission

*n* (%)	Japanese subgroup (*n* = 73)		Overall safety population (*N* = 689) [13]	
	Avelumab + BSC (*n* = 36)	BSC (*n* = 37)	Avelumab + BSC (*n* = 344)	BSC (*n* = 345)
Any TEAE	36 (100)	21 (56.8)	337 (98.0)	268 (77.7)
Grade ≥ 3 TEAE	18 (50.0)	3 (8.1)	163 (47.4)	87 (25.2)
Treatment-related TEAE	27 (75.0)	0	266 (77.3)	4 (1.2)
Grade ≥ 3 treatment-related TEAE	5 (13.9)	0	57 (16.6)	0
Serious TEAE	9 (25.0)	2 (5.4)	96 (27.9)	69 (20.0)
Serious treatment-related TEAE	5 (13.9)	0	31 (9.0)	0
TEAE leading to dose reduction of avelumab	0	–	1 (0.3)	–
TEAE leading to interruption of avelumab	16 (44.4)	–	140 (40.7)	–
TEAE leading to discontinuation of study drug	4 (11.1)	0	41 (11.9)	0
Treatment-related TEAE leading to discontinuation of study drug	4 (11.1)	0	33 (9.6)	0
TEAE leading to death	1 (2.8)	0	4 (1.2)	24 (7.0)
Treatment-related TEAE leading to death	1 (2.8)	0	1 (0.3)	0
Immune-related AE	13 (36.1)	2 (5.4)	101 (29.4)	5 (1.4)
Immune-related AE leading to discontinuation of study drug	1 (2.8)	0	19 (5.5)	0
Infusion-related reaction^a^	10 (27.8)	0	74 (21.5)	0

*AE* adverse event, *BSC* best supportive care, *TEAE* treatment-emergent adverse event

^a^Composite term including several prespecified preferred terms in addition to signs and symptoms of infusion-related reaction

**Table 6:** Most common TEAEs (related or unrelated to study treatment) in the Japanese subgroup and the overall population. Source: Data for the overall population have been published previously in Ref. [13]. Copyright © 2020 Massachusetts Medical Society. Reprinted with permission

Events, *n* (%)	Japanese subgroup (*n* = 73)				Overall safety population (*N* = 689) [13]			
	Avelumab + BSC (*n* = 36)		BSC (*n* = 37)		Avelumab + BSC (*n* = 344)		BSC (*n* = 345)	
	Any grade	Grade ≥ 3	Any grade	Grade ≥ 3	Any grade	Grade ≥ 3	Any grade	Grade ≥ 3
Any TEAE	36 (100)	18 (50.0)	21 (56.8)	3 (8.1)	337 (98.0)	163 (47.4)	268 (77.7)	87 (25.2)
Pyrexia	10 (27.8)	0	0	0	51 (14.8)	1 (0.3)	12 (3.5)	0
Anemia	7 (19.4)	4 (11.1)	1 (2.7)	1 (2.7)	39 (11.3)	13 (3.8)	23 (6.7)	10 (2.9)
Nasopharyngitis	7 (19.4)	0	5 (13.5)	0	26 (7.6)	0	13 (3.8)	0
Constipation	6 (16.7)	0	3 (8.1)	0	56 (16.3)	2 (0.6)	31 (9.0)	0
Nausea	6 (16.7)	0	0	0	54 (15.7)	1 (0.3)	22 (6.4)	2 (0.6)
Rash	6 (16.7)	0	0	0	40 (11.6)	1 (0.3)	4 (1.2)	0
Hypothyroidism	6 (16.7)	0	0	0	40 (11.6)	1 (0.3)	2 (0.6)	0
Vomiting	5 (13.9)	0	2 (5.4)	0	43 (12.5)	4 (1.2)	12 (3.5)	2 (0.6)
Hematuria	5 (13.9)	0	1 (2.7)	1 (2.7)	36 (10.5)	6 (1.7)	37 (10.7)	5 (1.4)
Infusion-related reaction	5 (13.9)	0	0	0	35 (10.2)	3 (0.9)	0	0
Cancer pain	4 (11.1)	1 (2.8)	2 (5.4)	0	8 (2.3)	1 (0.3)	6 (1.7)	1 (0.3)
Back pain	4 (11.1)	0	3 (8.1)	0	55 (16.0)	4 (1.2)	34 (9.9)	8 (2.3)
Diarrhea	4 (11.1)	0	0	0	57 (16.6)	2 (0.6)	17 (4.9)	1 (0.3)
Arthralgia	4 (11.1)	0	0	0	56 (16.3)	2 (0.6)	19 (5.5)	0
Headache	4 (11.1)	0	0	0	24 (7.0)	1 (0.3)	9 (2.6)	1 (0.3)
Hyperthyroidism	4 (11.1)	0	0	0	21 (6.1)	0	1 (0.3)	0
Stomatitis	4 (11.1)	0	1 (2.7)	0	7 (2.0)	0	1 (0.3)	0
Dental caries	4 (11.1)	0	0	0	4 (1.2)	0	0	0
WBC count decreased	4 (11.1)	0	0	0	4 (1.2)	0	0	0
Pyelonephritis	3 (8.3)	2 (5.6)	1 (2.7)	0	4 (1.2)	3 (0.9)	3 (0.9)	2 (0.6)
Urinary tract infection	3 (8.3)	1 (2.8)	1 (2.7)	0	59 (17.2)	15 (4.4)	36 (10.4)	9 (2.6)
Pruritus	3 (8.3)	0	1 (2.7)	0	59 (17.2)	1 (0.3)	6 (1.7)	0
Amylase increased	2 (5.6)	2 (5.6)	0	0	23 (6.7)	12 (3.5)	3 (0.9)	2 (0.6)
Blood triglycerides increased	2 (5.6)	2 (5.6)	0	0	3 (0.9)	3 (0.9)	0	0
Fatigue	2 (5.6)	1 (2.8)	1 (2.7)	0	61 (17.7)	6 (1.7)	24 (7.0)	2 (0.6)
Cough	2 (5.6)	0	2 (5.4)	0	44 (12.8)	1 (0.3)	16 (4.6)	0
Decreased appetite	1 (2.8)	0	4 (10.8)	0	47 (13.7)	1 (0.3)	23 (6.7)	2 (0.6)
Asthenia	0	0	0	0	56 (16.3)	0	19 (5.5)	4 (1.2)

Table shows TEAEs (preferred terms) occurring at any grade in ≥ 10% or grade ≥ 3 in ≥ 5% of patients in either arm in the Japanese subgroup or the overall population

*BSC* best supportive care, *TEAE* treatment-emergent adverse event, *WBC* white blood cell

In avelumab-treated patients, slight differences were seen in the most common TEAEs of any grade in Japanese patients compared with the overall population, including an increased occurrence of pyrexia (27.8% vs 14.8%) and nasopharyngitis (19.4% vs 7.6%) and a lower occurrence of fatigue (5.6% vs 17.7%), decreased appetite (2.8% vs 13.7%), and asthenia (0% vs 16.3%) (Table 6). The rate of discontinuation due to TEAEs in avelumab-treated patients was similar between the Japanese subgroup and the overall population (11.1% vs 11.9%).

Treatment-related AEs (TRAEs) of any grade with avelumab occurred in 75.0% of patients in the Japanese subgroup, including grade ≥ 3 TRAEs in 13.9% of patients (Online Resource 4). The most common TRAEs of any grade were hypothyroidism (16.7%), pyrexia (16.7%), infusion-related reaction (single term; 13.9%), hyperthyroidism (11.1%), and stomatitis (11.1%); the most common grade ≥ 3 TRAE was anemia (5.6%).

Using a composite term to identify infusion-related reactions (including several prespecified preferred terms in addition to signs and symptoms of infusion-related reaction), 10 patients (27.8%) had an infusion-related reaction (Table 5); all were grade 1/2.

In the Japanese subgroup, immune-related AEs (irAEs) of any grade occurred in 36.1% treated with avelumab, including grade 3 irAEs in 8.3%; no grade 4/5 irAEs occurred during the study (Online Resource 5). The irAEs comprised immune-related rash (19.4%), thyroid disorders (13.9%), pneumonitis (5.6%), and enteritis, adrenal insufficiency, vitiligo, and uveitis, each in 2.8%. Grade 3 irAEs comprised immune-related rash in 5.6% and enteritis in 2.8%. One Japanese patient (2.8%) discontinued avelumab because of an irAE (pneumonitis), compared with 5.5% discontinuing because of irAEs in the overall population.

Serious AEs occurred in 9 patients (25.0%) in the avelumab arm and in 2 patients (5.4%) in the control arm of the Japanese subgroup, including serious TRAEs in 5 patients (13.9%) in the avelumab arm (1 case each of gastric ulcer, vomiting, colitis, sepsis, anemia, decreased platelet count, interstitial lung disease, and myocardial infarction; some patients had ≥ 1 type of serious AE). One patient in the avelumab arm died following a TEAE (classified as treatment related); the patient had sepsis after a urinary tract infection and possible central venous catheter infection after receiving 11 infusions of avelumab, as reported previously [13].

## Discussion

Overall results from the JAVELIN Bladder 100 trial showed that avelumab 1L maintenance + BSC resulted in significantly longer OS than BSC alone in patients with advanced UC that had not progressed with 1L platinum-containing chemotherapy, both in the overall population and PD-L1 + population [13]. Findings from the Japanese subgroup were generally consistent with those in the overall population within the limitations of an underpowered exploratory subgroup analysis, further demonstrating the efficacy and safety of avelumab 1L maintenance.

Demographic and baseline characteristics in the Japanese subgroup were generally balanced between treatment arms. Minor differences between the total Japanese subgroup and the overall population included lower median weight, lower proportion with an ECOG PS ≥ 1, and higher proportion with an upper tract primary tumor or with baseline creatinine clearance < 60 mL/min. In addition, higher proportion of the Japanese subgroup had received gemcitabine + cisplatin as 1L chemotherapy and lower proportion had achieved an objective response with 1L chemotherapy. The duration of prior 1L chemotherapy was similar between Japanese patients and the overall population.

Among all Japanese patients, longer OS was observed with avelumab + BSC vs BSC alone (median OS, 24.7 vs 18.7 months; HR, 0.81 [95% CI, 0.41–1.58]). This occurred despite a higher proportion of Japanese patients receiving a subsequent anticancer drug therapy in the control arm (avelumab arm, 61.1%; control arm, 81.1%), including a PD-1 or PD-L1 inhibitor (38.9% vs 67.6%, respectively). Subsequent anticancer drug therapy was higher in Japanese patients than in the overall population (avelumab vs control in the overall population: any subsequent anticancer drug therapy, 42.3% vs 61.7%; subsequent PD-1/PD-L1 inhibitor, 6.3% vs 43.7%), which may have led to longer OS in the Japanese subgroup. These data show that subsequent PD-1/PD-L1 inhibitor use after avelumab was also higher in Japanese patients than in the overall population. In Japanese patients with PD-L1 + tumors, median OS was similar in both arms; however, 95% CIs were wide because of the small numbers of patients and events. In addition, some differences were observed in demographic and disease characteristics between the treatment arms in Japanese patients with PD-L1 + tumors, which may have affected OS outcomes. Longer PFS was observed in the avelumab arm vs the control arm among Japanese patients, both in the overall subgroup (median, 5.6 vs 1.9 months; HR, 0.63 [95% CI, 0.36–1.11]) and in those with PD-L1 + tumors (median, 5.6 vs 1.9 months; HR, 0.62 [95% CI, 0.30–1.30]).

The safety profile of avelumab in Japanese patients was consistent with that of the overall population, and no safety concerns specific to Japanese patients were identified. Slight differences were seen in the most common TEAEs of any grade in Japanese patients treated with avelumab compared with the overall population. However, because the median relative dose intensity of avelumab was similar between Japanese patients and the overall population (87.8% vs 88.2%), these observations might be due to ethnic differences or sampling constraints in a smaller patient group. No notable differences in the occurrence of irAEs were observed between Japanese patients and the overall population (36.1% vs 29.4%), and rates of discontinuation due to TEAEs (11.1% vs 11.9%) or irAEs (2.8% vs 5.5%) were low in both groups.

In conclusion, avelumab 1L maintenance treatment showed a favorable benefit-risk balance in Japanese patients, similar to that in the overall population. These results support avelumab 1L maintenance as a new standard of care in Japanese patients with advanced UC that has not progressed with 1L platinum-containing chemotherapy.

## Supplementary Information

Below is the link to the electronic supplementary material.

Supplementary file1 (DOCX 44 KB)

## Data Availability

Upon request, and subject to certain criteria, conditions and exceptions (see https://www.pfizer.com/science/clinical-trials/trial-data-and-results for more information), Pfizer will provide access to individual de-identified participant data from Pfizer-sponsored global interventional clinical studies conducted for medicines, vaccines and medical devices (1) for indications that have been approved in the US and/or EU or (2) in programs that have been terminated (ie, development for all indications has been discontinued). Pfizer will also consider requests for the protocol, data dictionary, and statistical analysis plan. Data may be requested from Pfizer trials 24 months after study completion. The de-identified participant data will be made available to researchers whose proposals meet the research criteria and other conditions, and for which an exception does not apply, via a secure portal. To gain access, data requestors must enter into a data access agreement with Pfizer.
