# Supplementary material for: Avelumab first-line maintenance plus best supportive care (BSC) vs BSC alone for advanced urothelial carcinoma: JAVELIN Bladder 100 Japanese subgroup analysis
Source: Int J Clin Oncol. 2022 Jan 1;27(2):383–95. doi: 10.1007/s10147-021-02067-8 (PMC8816770; doi:10.1007/s10147-021-02067-8)
Supplement: Supplementary file 1 — Supplementary file1 (DOCX 44 KB) [file 10147_2021_2067_MOESM1_ESM.docx]

**ONLINE RESOURCES**

**Avelumab first-line maintenance plus best supportive care (BSC) vs BSC alone for advanced urothelial carcinoma: JAVELIN Bladder 100 Japanese subgroup analysis**

*International Journal of Clinical Oncology*

Yoshihiko Tomita, Yoshiaki Yamamoto, Norihiko Tsuchiya, Hiroomi Kanayama, Masatoshi Eto, Hideaki Miyake, Thomas Powles, Mizuki Yoshida, Yuichiro Koide, Yoshiko Umeyama, Alessandra di Pietro, Hirotsugu Uemura

**Corresponding author**

Yoshihiko Tomita, MD, PhD

Departments of Urology and Molecular Oncology, Niigata University Graduate School of Medicine, Niigata 951-8510, Japan

Email: [ytomita@med.niigata-u.ac.jp](mailto:ytomita@med.niigata-u.ac.jp)

Phone: +81-25-227-2289

Fax: +81-25-227-0784

**Online Resource 1** Patient disposition at data cutoff (October 21, 2019) in the Japanese subgroup and the overall population

| **n (%)** | **Japanese subgroup** | | | | **Overall population** | | | |
| --- | --- | --- | --- | --- | --- | --- | --- | --- |
|  | **Randomized patients  (n=73)** | | **PD-L1+ population  (n=42)** | | **Randomized patients (N=700)** | | **PD-L1+ population  (N=358)** | |
|  | **Avelumab + BSC (n=36)** | **BSC (n=37)** | **Avelumab + BSC**  **(n=19)** | **BSC (n=23)** | **Avelumab + BSC (n=350)** | **BSC (n=350)** | **Avelumab + BSC (n=189)** | **BSC (n=169)** |
| **Ongoing** | 7 (19.4) | 2 (5.4) | 5 (26.3) | 2 (8.7) | 85 (24.3) | 26 (7.4) | 58 (30.7) | 13 (7.7) |
| **Discontinued** | 29 (80.6) | 35 (94.6) | 14 (73.7) | 21 (91.3) | 265 (75.7) | 324 (92.6) | 131 (69.3) | 156 (92.3) |
| Progressive disease | 21 (58.3) | 28 (75.7) | 10 (52.6) | 20 (87.0) | 189 (54.0) | 263 (75.1) | 84 (44.4) | 126 (74.6) |
| Adverse event | 4 (11.1) | 0 | 2 (10.5) | 0 | 39 (11.1) | 2 (0.6) | 26 (13.8) | 1 (0.6) |
| Death | 2 (5.6) | 0 | 1 (5.3) | 0 | 5 (1.4) | 14 (4.0) | 3 (1.6) | 8 (4.7) |
| Physician's decision | 1 (2.8) | 0 | 1 (5.3) | 0 | 5 (1.4) | 7 (2.0) | 4 (2.1) | 6 (3.6) |
| Withdrawal by patient | 1 (2.8) | 6 (16.2) | 0 | 1 (4.3) | 16 (4.6) | 29 (8.3) | 7 (3.7) | 12 (7.1) |
| Global deterioration of health status | 0 | 1 (2.7) | 0 | 0 | 4 (1.1) | 6 (1.7) | 2 (1.1) | 1 (0.6) |
| Noncompliance with study drug | 0 | 0 | 0 | 0 | 1 (0.3) | 0 | 1 (0.5) | 0 |
| No longer meets eligibility criteria | 0 | 0 | 0 | 0 | 3 (0.9) | 0 | 1 (0.5) | 0 |
| Lost to follow-up | 0 | 0 | 0 | 0 | 2 (0.6) | 2 (0.6) | 2 (1.1) | 1 (0.6) |
| Other | 0 | 0 | 0 | 0 | 1 (0.3) | 1 (0.3) | 1 (0.5) | 1 (0.6) |

BSC, best supportive care; PD-L1, programmed death ligand 1.

**Online Resource 2** Subsequent anticancer drug therapies in the Japanese subgroup and the overall population

| **n (%)** | **Japanese subgroup (n=73)** | | **Overall population (N=700)** | |
| --- | --- | --- | --- | --- |
|  | **Avelumab + BSC (n=36)** | **BSC (n=37)** | **Avelumab + BSC (n=350)** | **BSC (n=350)** |
| **Number of subsequent anticancer drug regimens**  0  1  2  3  ≥4  Not reported | 4 (11.1)  10 (27.8)  8 (22.2)  3 (8.3)  1 (2.8)  10 (27.8) | 4 (10.8)  14 (37.8)  11 (29.7)  3 (8.1)  2 (5.4)  3 (8.1) | 73 (20.9) 102 (29.1)  33 (9.4)  11 (3.1)  2 (0.6)  129 (36.9) | 50 (14.3)  150 (42.9)  52 (14.9)  11 (3.1)  3 (0.9)  84 (24.0) |
| **Any subsequent anticancer drug therapy**  Pembrolizumab  Gemcitabine  Carboplatin  Cisplatin  Paclitaxel  Docetaxel  Ifosfamide  Nedaplatin  Atezolizumab  Vinflunine  Nivolumab  Durvalumab | 22 (61.1)  14 (38.9)  13 (36.1)  11 (30.6)  9 (25.0)  5 (13.9)  1 (2.8)  0  0  0  0  0  0 | 30 (81.1)  24 (64.9)  16 (43.2)  8 (21.6)  11 (29.7)  6 (16.2)  3 (8.1)  2 (5.4)  2 (5.4)  1 (2.7)  0  0  0 | 148 (42.3)  19 (5.4)  61 (17.4)  46 (13.1)  29 (8.3)  48 (13.7)  5 (1.4)  0  0  3 (0.9)  37 (10.6)  0  0 | 216 (61.7)  71 (20.3)  52 (14.9)  36 (10.3)  21 (6.0)  41 (11.7)  10 (2.9)  2 (0.6)  2 (0.6)  49 (14.0)  17 (4.9)  18 (5.1)  16 (4.6) |

BSC, best supportive care.

Table shows individual drug therapies received by ≥3% of patients in either arm in the Japanese subgroup or the overall population.

**Online Resource 3** Exposure to study drugs in the Japanese subgroup and the overall population

|  | **Japanese subgroup (n=73)** | | **Overall safety population (N=689)** | |
| --- | --- | --- | --- | --- |
|  | **Avelumab + BSC (n=36)** | **BSC (n=37)** | **Avelumab +BSC (n=344)** | **BSC (n=345)** |
| Duration of treatment, median (range), weeks | 32.0 (2.0-159.9) | 9.1 (0.1-90.0) | 24.9 (2.0-159.9) | 13.1 (0.1-155.6) |
| Dose intensity, median (range), mg/kg/4-week cycle | 17.6 (10.0-19.8) | – | 17.6 (1.6-20.4) | – |
| Relative dose intensity, median (range), % | 87.8 (50.0-99.1) | – | 88.2 (8.0-102.1) | – |
| Patients with ≥1 dose delay, n (%) | 16 (44.4) | – | 158 (45.9) | – |
| Patients with ≥1 dose reduction, n (%) | 0 | – | 11 (3.2) | – |
| Patients with ≥1 infusion-rate reduction of ≥50%, n (%) | 7 (19.4) | – | 32 (9.3) | – |
| Patients with ≥1 infusion interruption, n (%) | 3 (8.3) | – | 16 (4.7) | – |

BSC, best supportive care.

Dose intensity (mg/kg/4-week cycle) = [overall cumulative dose (mg/kg)]/[intended duration of avelumab treatment (weeks)/4].

Relative dose intensity (%) = 100 × [dose intensity (mg/kg/4-week cycle)]/[20 (mg/kg/4-week cycle)].

Dose reduction is defined as the actual nonzero dose <90% of the planned dose.

**Online Resource 4** Summary of the most common TRAEs in the Japanese subgroup and the overall population

| **n (%)** | **Japanese subgroup (n=73)** | | | | **Overall safety population (N=689)** | | | |
| --- | --- | --- | --- | --- | --- | --- | --- | --- |
|  | **Avelumab + BSC (n=36)** | | **BSC (n=37)** | | **Avelumab + BSC (n=344)** | | **BSC (n=345)** | |
|  | **Any grade** | **Grade ≥3** | **Any grade** | **Grade ≥3** | **Any grade** | **Grade ≥3** | **Any grade** | **Grade ≥3** |
| **Any TRAE** | 27 (75.0) | 5 (13.9) | 0 | 0 | 266 (77.3) | 57 (16.6) | 4 (1.2) | 0 |
| Hypothyroidism | 6 (16.7) | 0 | 0 | 0 | 36 (10.5) | 1 (0.3) | 0 | 0 |
| Pyrexia | 6 (16.7) | 0 | 0 | 0 | 23 (6.7) | 0 | 0 | 0 |
| Infusion-related reaction | 5 (13.9) | 0 | 0 | 0 | 35 (10.2) | 3 (0.9) | 0 | 0 |
| Hyperthyroidism | 4 (11.1) | 0 | 0 | 0 | 21 (6.1) | 0 | 0 | 0 |
| Stomatitis | 4 (11.1) | 0 | 0 | 0 | 5 (1.5) | 0 | 0 | 0 |
| Anemia | 3 (8.3) | 2 (5.6) | 0 | 0 | 11 (3.2) | 5 (1.5) | 0 | 0 |
| Neutrophil count decreased | 3 (8.3) | 1 (2.8) | 0 | 0 | 7 (2.0) | 1 (0.3) | 0 | 0 |
| Diarrhea | 3 (8.3) | 0 | 0 | 0 | 35 (10.2) | 0 | 0 | 0 |
| Chills | 3 (8.3) | 0 | 0 | 0 | 24 (7.0) | 0 | 0 | 0 |
| Blood creatine phosphokinase increased | 2 (5.6) | 1 (2.8) | 0 | 0 | 11 (3.2) | 3 (0.9) | 0 | 0 |
| Platelet count decreased | 2 (5.6) | 1 (2.8) | 0 | 0 | 4 (1.2) | 1 (0.3) | 0 | 0 |
| Vomiting | 2 (5.6) | 0 | 0 | 0 | 14 (4.1) | 1 (0.3) | 0 | 0 |
| Rash maculopapular | 2 (5.6) | 0 | 0 | 0 | 12 (3.5) | 1 (0.3) | 0 | 0 |
| Aspartate aminotransferase increased | 2 (5.6) | 0 | 0 | 0 | 8 (2.3) | 2 (0.6) | 0 | 0 |
| Edema peripheral | 2 (5.6) | 0 | 0 | 0 | 8 (2.3) | 0 | 0 | 0 |
| Dermatitis | 2 (5.6) | 0 | 0 | 0 | 7 (2.0) | 0 | 0 | 0 |
| WBC count decreased | 2 (5.6) | 0 | 0 | 0 | 2 (0.6) | 0 | 0 | 0 |
| Lipase increased | 1 (2.8) | 1 (2.8) | 0 | 0 | 13 (3.8) | 10 (2.9) | 0 | 0 |
| Hypophosphatemia | 1 (2.8) | 1 (2.8) | 0 | 0 | 4 (1.2) | 2 (0.6) | 0 | 0 |
| Erythema multiforme | 1 (2.8) | 1 (2.8) | 0 | 0 | 1 (0.3) | 1 (0.3) | 0 | 0 |
| Gastric ulcer | 1 (2.8) | 1 (2.8) | 0 | 0 | 1 (0.3) | 1 (0.3) | 0 | 0 |
| Pancreatic enzyme abnormality | 1 (2.8) | 1 (2.8) | 0 | 0 | 1 (0.3) | 1 (0.3) | 0 | 0 |
| Sepsis | 1 (2.8) | 1 (2.8) | 0 | 0 | 1 (0.3) | 1 (0.3) | 0 | 0 |
| Pruritus | 1 (2.8) | 0 | 0 | 0 | 47 (13.7) | 1 (0.3) | 0 | 0 |
| Fatigue | 1 (2.8) | 0 | 0 | 0 | 33 (9.6) | 1 (0.3) | 0 | 0 |
| Rash | 1 (2.8) | 0 | 0 | 0 | 25 (7.3) | 1 (0.3) | 0 | 0 |
| Nausea | 1 (2.8) | 0 | 0 | 0 | 24 (7.0) | 1 (0.3) | 0 | 0 |
| Dry skin | 1 (2.8) | 0 | 0 | 0 | 18 (5.2) | 0 | 0 | 0 |
| Asthenia | 0 | 0 | 0 | 0 | 34 (9.9) | 0 | 0 | 0 |
| Arthralgia | 0 | 0 | 0 | 0 | 23 (6.7) | 1 (0.3) | 0 | 0 |
| Amylase increased | 0 | 0 | 0 | 0 | 15 (4.4) | 7 (2.0) | 0 | 0 |

BSC, best supportive care; TRAE, treatment-related adverse event; WBC, white blood cell.

Table shows TRAEs (preferred terms) occurring at any grade in ≥5% of patients or grade ≥3 in ≥2% of patients in either arm in the Japanese subgroup or the overall population.

Source: Data for the overall population have been published previously in New England Journal of Medicine, Powles T, et al, Avelumab maintenance therapy for advanced or metastatic urothelial carcinoma, Volume 383, Pages 1218-1230. Copyright © 2020 Massachusetts Medical Society. Reprinted with permission.

**Online Resource 5** Summary of irAEs in the avelumab arm of the Japanese subgroup and the overall population

| **n (%)** | **Japanese subgroup (n=36)** | | **Overall safety population (n=344)** | |
| --- | --- | --- | --- | --- |
|  | **Any grade** | **Grade ≥3** | **Any grade** | **Grade ≥3** |
| **Any irAE** | 13 (36.1) | 3 (8.3) | 101 (29.4) | 24 (7.0) |
| **Immune-related rash** | 7 (19.4) | 2 (5.6) | 35 (10.2) | 5 (1.5) |
| Rash | 2 (5.6) | 0 | 17 (4.9) | 1 (0.3) |
| Drug eruption | 1 (2.8) | 1 (2.8) | 1 (0.3) | 1 (0.3) |
| Erythema multiforme | 1 (2.8) | 1 (2.8) | 1 (0.3) | 1 (0.3) |
| Rash maculopapular | 1 (2.8) | 0 | 8 (2.3) | 1 (0.3) |
| Erythema | 1 (2.8) | 0 | 2 (0.6) | 1 (0.3) |
| Purpura | 1 (2.8) | 0 | 2 (0.6) | 0 |
| Rash erythematous | 1 (2.8) | 0 | 2 (0.6) | 0 |
| Lichen planus | 1 (2.8) | 0 | 1 (0.3) | 0 |
| Rash papular | 1 (2.8) | 0 | 1 (0.3) | 0 |
| Pruritus | 0 | 0 | 7 (2.0) | 0 |
| Rash pruritic | 0 | 0 | 1 (0.3) | 0 |
| **Immune-related thyroid disorders** | 5 (13.9) | 0 | 42 (12.2) | 1 (0.3) |
| Hypothyroidism | 5 (13.9) | 0 | 35 (10.2) | 1 (0.3) |
| Hyperthyroidism | 2 (5.6) | 0 | 16 (4.7) | 0 |
| Autoimmune thyroiditis | 0 | 0 | 2 (0.6) | 0 |
| Autoimmune hypothyroidism | 0 | 0 | 1 (0.3) | 0 |
| Blood thyroid-stimulating hormone increased | 0 | 0 | 1 (0.3) | 0 |
| Thyroiditis | 0 | 0 | 1 (0.3) | 0 |
| Thyroxine free decreased | 0 | 0 | 1 (0.3) | 0 |
| **Immune-related pneumonitis** | 2 (5.6) | 0 | 7 (2.0) | 1 (0.3) |
| Pneumonitis | 1 (2.8) | 0 | 5 (1.5) | 1 (0.3) |
| Interstitial lung disease | 1 (2.8) | 0 | 2 (0.6) | 0 |
| **Immune-related colitis** | 1 (2.8) | 1 (2.8) | 5 (1.5) | 3 (0.9) |
| Enteritis | 1 (2.8) | 1 (2.8) | 1 (0.3) | 1 (0.3) |
| Colitis | 0 | 0 | 3 (0.9) | 2 (0.6) |
| Diarrhea | 0 | 0 | 2 (0.6) | 0 |
| Proctitis | 0 | 0 | 1 (0.3) | 0 |
| **Immune-related adrenal insufficiency** | 1 (2.8) | 0 | 3 (0.9) | 0 |
| Adrenal insufficiency | 1 (2.8) | 0 | 3 (0.9) | 0 |
| **Immune-related AE: other** | 1 (2.8) | 0 | 9 (2.6) | 2 (0.6) |
| Vitiligo | 1 (2.8) | 0 | 2 (0.6) | 0 |
| Psoriasis | 0 | 0 | 3 (0.9) | 0 |
| Oligoarthritis | 0 | 0 | 1 (0.3) | 1 (0.3) |
| Rheumatoid arthritis | 0 | 0 | 1 (0.3) | 1 (0.3) |
| Arthritis | 0 | 0 | 1 (0.3) | 0 |
| Dermatitis psoriasiform | 0 | 0 | 1 (0.3) | 0 |
| Polyarthritis | 0 | 0 | 1 (0.3) | 0 |
| **Immune-related AE: uveitis** | 1 (2.8) | 0 | 1 (0.3) | 0 |
| Uveitis | 1 (2.8) | 0 | 1 (0.3) | 0 |
| **Immune-related type 1 diabetes mellitus** | 0 | 0 | 3 (0.9) | 3 (0.9) |
| Hyperglycemia | 0 | 0 | 3 (0.9) | 3 (0.9) |
| Diabetes mellitus | 0 | 0 | 0 | 0 |
| **Immune-related nephritis and**  **renal dysfunction** | 0 | 0 | 6 (1.7) | 1 (0.3) |
| Renal failure | 0 | 0 | 3 (0.9) | 1 (0.3) |
| Nephritis | 0 | 0 | 3 (0.9) | 0 |
| Tubulointerstitial nephritis | 0 | 0 | 1 (0.3) | 0 |
| **Immune-related hepatitis** | 0 | 0 | 5 (1.5) | 5 (1.5) |
| Alanine aminotransferase increased | 0 | 0 | 3 (0.9) | 3 (0.9) |
| Aspartate aminotransferase increased | 0 | 0 | 2 (0.6) | 2 (0.6) |
| Autoimmune hepatitis | 0 | 0 | 1 (0.3) | 1 (0.3) |
| Hepatotoxicity | 0 | 0 | 1 (0.3) | 1 (0.3) |
| **Immune-related pancreatitis** | 0 | 0 | 2 (0.6) | 1 (0.3) |
| Autoimmune pancreatitis | 0 | 0 | 1 (0.3) | 1 (0.3) |
| Pancreatitis | 0 | 0 | 1 (0.3) | 0 |
| **Immune-related myositis** | 0 | 0 | 2 (0.6) | 2 (0.6) |
| Myositis | 0 | 0 | 2 (0.6) | 2 (0.6) |
| **Immune-related Guillain-Barré syndrome** | 0 | 0 | 1 (0.3) | 1 (0.3) |
| Miller Fisher syndrome | 0 | 0 | 1 (0.3) | 1 (0.3) |

AE, adverse event; irAE, immune-related adverse event.

Source: Data for the overall population have been published previously in New England Journal of Medicine, Powles T, et al, Avelumab maintenance therapy for advanced or metastatic urothelial carcinoma, Volume 383, Pages 1218-1230. Copyright © 2020 Massachusetts Medical Society. Reprinted with permission.
